# New and Non-Official Remedies

**Published:** 1911-12

**Authors:** 


### New and Non-Official Remedies

### FORMIC ACID AND ALLIED COMPOUNDS.

Formic acid (24.25 per cent. of absolute acid). While considered analogous to acetic acid as irritant and antiseptic, but "more so," the Council deprecate its internal use. Externally, it is counter irritant, 1% in alcohol or alcohol and water. By this method, it is a more controlable substitute for the time-honored bee-sting method.

Sodium formate. (See above) 10—25 centigrams (1½—4 grains.)

Methylene dicotoin (Fortoin). antiseptic, especially in putrefactive diarrhoea. 25 centigrams (4 grains).

Formalin gelatin (Glutol) dusting powder.

Trioxymethylene (Paraformaldehyde) is a polymeric condensation of formaldehyde, in powder form. It is generally used to prepare formaldehyde by heating but may be used internally, 1-3 —1 gram (5—15 grains) well diluted or in 10% suspension in collodion as an escharotic for warts, etc.

### NUCLEIN AND ALLIED SUBSTANCES.

Nuclein ½—1 gram (7½—15 grains).

Nucleic acid 6—30 centigrams (1—5 grains).

Sodium nucleate same dose.

### DIETHYL BARBITURIC COMPOUNDS.

Diethyl-barbituric acid, (Veronal) hypnotic 1-3—1 gram (5—15 grains).

Sodium diethyl-barbiturate (Medinal) (Veronal-sodium) same dose.

### TANNIN COMPOUNDS.

Albumin tannate (Tannalbin) 1—4 grams (15—60 grains).

Tannyl acetate (Tannigen) 20—60 centigrams (3—10 grains).

Bismuth bitannate (Tannismuth) 30—60 centigrams (5—10 grains).

Tannin formaldehyde (Tannoform) 25—50 centigrams (4—7½ grains), externally full strength or diluted with talc to half or quarter strength or 10% in ointment or soap.

Hexamethylene-tetramine-tannin (Tannopin) 30—50 centigrams, (5—7½ grains).

Ellagic acid (Gallogen) 30—50 centigrams (5—7½ grains).

Eucalyptus gum (Red gum) 10—30 centigrams (1½—5 grains) in trochee.

Tannin nucleo-proteid (Protan) 50% combination of casein and tannin. 3 centigrams to 2 grams (½—30 grains) according to age, frequency of dosage and degree of diarrhoea.

VALERIC ACID COMPOUNDS.

Amyl valerate especially for biliary colic, 20—40 centigrams (3—6 minims) every half hour or 1 c.c. (15 minims) t.i.d.

Borneol valerate (Bornyval) actually forms a large part of natural oil of valerian. 25—75 centigrams (4—12 grains).

Menthyl valerate. (Validol is menthyl valerate plus 30% of free menthol) gtt. x—xv.

Validol camphoratum, Validol plus 10% camphor, same dose.

Diethylamine valerate (Valyl) supplied in pearls, owing to oxidation in air. Dose 2—3 pearls of 2 grains each.

COCAINE ANALOGUES.

(Alpha-eucaine) triacetonamine derivative, withdrawn from market.

(Beta-eucaine) tri-methyl-benzo-oxypiperadin. Prepared as hydrochlorid and lactate, the latter more soluble. Analogous to cocaine but neither mydriatic nor vaso-contractor and stable even on boiling. 1—10% in instillation, injection, ointment, etc., according to location &c.

Holocaine hydrochlorid, is phenitidyl-acetphenitidin hydrochlorid. Quicker than cocaine, with added antiseptic action and said not to produce scaliness of cornea. 1%.

Homatropine hydrochlorid and hydrobromid mainly used as mydriatics. 1%.

Methylatropine nitrate (Eumydrin). Less toxic than atropine, and as mydriatic, prompter and less prolonged action. Slightly larger dose and stronger solution than atropine.

Euphthalmin. (hydrochlorid) Mydriatic without anesthetic action and with very little action on accommodation and tension. 5—10%. General action similar to atropine.

Tropacocaine hydrochlorid. local anesthetic, prompter and more prolonged action and less toxicity than from cocaine, less mydriatic. 3—10%.

(Novacaine) hydrochlorid and nitrate (latter for combination with silver) less toxic and prompter than cocaine, but less sustained unless combined with adrenalin. ¼—20% solutions, according to area anaesthetized, etc. Internal dose up to ½ gram (7½ grains).

(Alypin) Local anaesthetic, without mydriasis or disturbance of accommodation. 1—2% in eye; 1—4% for injection anaesthesia; 10% externally.

(Stovaine) similar to alypin. 2 milligrams (1-30 grain) internally; up to 4% in eye; 5—10% on mucous membranes; 1% for injection anaesthesia.

OTHER LOCAL ANAESTHETICS.

(Anaesthesin.) Ethyl paramido-benzoate. 30—50 centigrams (5—7½ grains) in gastric ulcer, hyperaesthesia and vomiting or in suppositories. 4—10% ointment or dusting powder; 3% spray up to 50% local application on mucus membranes.

Ethyl amino-benzoate. Insoluble, otherwise used similarly to above.

(Orthoform New) Insoluble, similar to above.

(Orthoform New, hydrochlorid) soluble 10% in water.

